# Tissue factor-targeted immunotherapy of melanoma and triple negative breast cancer using a second generation ICON

**DOI:** 10.1186/2051-1426-3-S2-P304

**Published:** 2015-11-04

**Authors:** Zhiwei Hu, Elizabeth McMichael, Amanda Campbell, Cheryl A London, William E Carson

**Affiliations:** 1 The Ohio State University, Columbus, OH, USA

## Background

**Figure 1:**
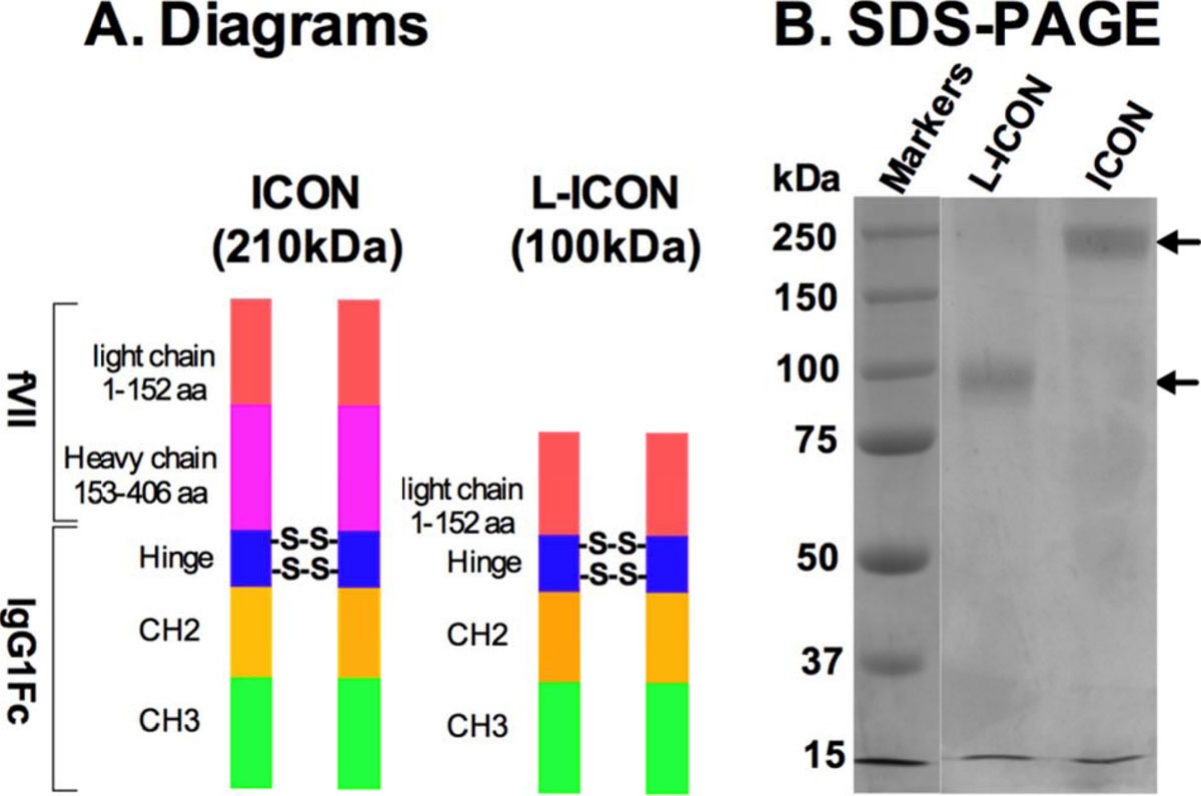


Hu and colleagues have identified tissue factor (TF-the primary initiator of coagulation and a modulator of angiogenesis) as a common yet specific biomarker and therapeutic target on a variety of cancer cells and angiogenic tumor vascular endothelial cells [1-3]. He has co-invented a TF-targeting Immuno-Conjugate agent named ICON that consists of factor VII (1-406 aa, the natural ligand to tissue factor) fused to the Fc region of IgG1 [1, 2, 4, 5]. Intra-lesional ICON therapy of experimental murine melanoma tumors with an adenoviral vector leads to marked growth tumor inhibition with minimal effects on normal tissues [1]. However, ICON has a relatively big molecular weight (210 kDa) (Figure 1) [5]. To reduce its molecular mass, Hu has developed a second generation ICON, named L-ICON, which consists of only the light chain (1-152 aa) of fVII fused to IgG1Fc (Figure 1). This proposal is designed to evaluate the effects of L-ICON immunotherapy for the treatment of BRAF mutated melanoma and triple negative breast cancer (TNBC), both of which are very difficult to treat in clinic.

## Methods

L-ICON protein and adenoviral vectors have been developed by recombinant DNA techniques. The binding activity of L-ICON to human melanoma lines with or without BRAF mutant and to human TNBC was assayed by flow cytometry and cell ELISA. Its ADCC effect was determined by an ADCC effector assay (Promega). L-ICON therapy of TNBC via intra-lesional injection of Ad-L-ICON was compared to Ad-ICON in a nude mouse model of TNBC MDA-MB-231.

## Results

The molecular weight of L-ICON was 100 kDa (Fig. 1), only 50% of ICON's. L-ICON could bind to human and canine melanoma lines, regardless of their BRAF status. L-ICON could also bind to human TNBC lines, similarly to the first generation ICON. L-ICON could mediate ADCC effect to these cancer cells *in vitro*. Intra-lesional L-ICON and ICON immunotherapy via adenoviral vectors were similarly effective for the treatment of human TNBC in a nude mouse model.

## Conclusions

L-ICON molecular mass was reduced significantly while its binding activity to cancer cells was intact. L-ICON therapy was effective for the treatment of melanoma and TNBC *in vitro* and *in vivo* in a preclinical mouse model.
